# Positive Correlation Between LTA Expression and Overall Immune Activity Suggests an Increased Probability of Survival in Uterine Corpus Endometrial Carcinoma

**DOI:** 10.3389/fcell.2021.793793

**Published:** 2022-01-28

**Authors:** Mingjie Shi, Fei Luo, Taotao Shao, Hengli Zhang, Taili Yang, Yue Wei, Riling Chen, Runmin Guo

**Affiliations:** ^1^ Key Laboratory of Research in Maternal and Child Medicine and Birth Defects, Guangdong Medical University, Foshan, China; ^2^ Matenal and Child Research Institute, Shunde Women and Children’s Hospital of Guangdong Medical University (Maternity and Child Healthcare Hospital of Shunde Foshan), Foshan, China; ^3^ First College of Clinical Medicine, Guangdong Medical University, Zhanjiang, China; ^4^ Department of Ultrasound, Shunde Women and Children’s Hospital of Guangdong Medical University (Maternity and Child Healthcare Hospital of Shunde Foshan), Foshan, China

**Keywords:** endometrial carcinoma, immune activity, prognosis, tumor microenvironment, immunotherapy

## Abstract

Mounting evidence indicates that immune status plays a crucial role in tumor progress and metastasis, while there are no effective and easily assayed biomarkers to reflect it in uterine corpus endometrial carcinoma (UCEC) patients. Here, we attempted to identify the potential biomarkers that were differentially expressed between normal and tumor tissues and involved in prognosis and immune microenvironment of UCEC patients. RNA-seq data with relevant clinical information were obtained from The Cancer Genome Atlas (TCGA). ssGSEA algorithm was applied to calculate the enrichment scores of every tumor infiltration lymphocyte (TIL) set in each sample, and patients were then divided into three clusters using multiple R packages. Cox analysis, ESTIMATE, and CIBERSORT were utilized to determine the differentially expressed immune-related genes (DEIGs) with overall survival, and to explore their roles in prognosis, immune microenvironment, and immunotherapeutic response. The TIMER and TISIDB databases were utilized to predict the effectiveness of immunotherapy in UCEC patients. LTA was finally identified to be significantly upregulated in tumor tissues and closely associated with prognosis and immunological status, which was then verified in GSE17025. In multivariate analysis, the hazard ratio of LTA was 0.42 with 95% CI (0.22–0.80) (*p* = 0.008). Patients with high LTA expression had better survival and apparently immune-activated phenotypes, such as more tumor mutation burden (TMB), stronger immune cell infiltrations, higher expression of immunosuppressive points, and higher immunophenoscore, meaning they had an immunotherapeutic advantage over those with low LTA expression. TIMER and TISIDB indicated that LTA was highly expressed in UCEC, and its expression was negatively correlated with stages and positively related to prognosis. Additionally, we found that LTA ectopic expression weakened the proliferation ability of RL95-2 cells. All these findings indicated that LTA could act as a novel and easily assayed biomarker to predict immunological status and clinical outcomes and even as an antioncogene to explore UCEC in depth.

## Introduction

Uterine corpus endometrial carcinoma (UCEC) is a common malignancy in females (Bray et al., 2018). The overall 5-year survival rate of patients with early-stage or localized lesions could achieve 75%–86% by performing surgery, while the survival time of patients with metastasis or recurrence might drop below 16 weeks (Chaudhry and Asselin, 2009; Gottwald et al., 2010; Lortet-Tieulent et al., 2018). Additionally, the treatment regimens are mainly devised based on the histopathologic stage of patients, but there are insufficient considerations about individual differences (Brooks et al., 2019). Recently, immunotherapy is a new breakthrough in cancer treatment for its safety and sustainability, and immune status, including tumor microenvironment (TME), tumor mutation burden (TMB), and the expression of immune checkpoints, was an indicator to evaluate the likelihood of tumor patients benefiting from it. Patients with the following characteristics can also be considered to have a high probability of benefiting from immunotherapy, such as microsatellite instability (Fujimoto et al., 2020), more abundance of DNA polymerase *ϵ* (POLE) mutation (Hwang et al., 2021), and higher levels of immunosuppressants targets (Paré et al., 2018). However, they are unsuitable as desirable markers for dynamically monitoring the immune status and prognosis of patients because of the difficulty in evaluation.

Increasing studies demonstrated that immune cell infiltrations and microenvironment were closely related to tumor development, metastasis, therapeutic response, and prognosis (Galon et al., 2013; Pagès et al., 2018; Yu et al., 2018). The proportion of immune components in TME and TMB could both affect their immune phenotypes, thereby influencing tumor prognosis and therapeutic efficacy (Binnewies et al., 2018; Gao et al., 2021; Ji et al., 2021). Thus, identifying the regulatory molecules between them may facilitate providing novel and effective therapeutics for UCEC patients. Single-sample GSEA (ssGSEA), a novel algorithm, has been applied to calculate separate enrichment scores for each pairing of a sample and gene set, which are applied to assess the abundance of immune components and predict the clinical outcomes of patients. Here, we performed a comprehensive analysis based on the transcriptome profiling and corresponding clinic data of UCEC subjects from The Cancer Genome Atlas (TCGA). ssGSEA algorithm was utilized to calculate the enrichment scores of every tumor infiltration lymphocyte (TIL) gene set in each UCEC patient. Patients were divided into three immune-related clusters based on them by several R packages; we extracted shared differentially expressed immune-related genes (DEIGs) and identified those DEIGs associated with the prognosis of UCEC patients. The differences in clinical characteristics and immune phenotypes (such as immune infiltrations, immune checkpoint expressions, and immunophenoscore) in UCEC tumor patients were then evaluated by multiple bioinformatics methods (Supplementary Figure S1).

## Materials and Methods

### Download and Processing of the UCEC Dataset

Transcriptome RNA sequences (FPKM value) and relevant clinical information of UCEC patients in TCGA (which contained 23 normal subjects and 548 tumor cases) were both downloaded from the UCSC Xena database (https://portal.gdc.cancer.gov/). We extracted sample data according to the following criteria: (1) removing duplicated samples involved in paraffin-embedded and formalin-fixed, (2) deleting patients with insufficient clinical data, and (3) abstracting the average of duplicated genes with the same ensemble ID. A total of 521 patients with UCEC in TCGA were incorporated into our study, and the basic information of subjects in the study is shown in Table 1. GSE17025, including 12 normal samples and 91 early-stage UCEC samples, was collected from the GEO database (http://www.ncbi.nlm.nih.gov/geo) based on the GPL570 (Affymetrix Human Genome U133 Plus 2.0 Array) platform. The Limma package (http://bioconductor.org/packages/release/bioc/html/limma.html) was applied to identify differentially expressed genes using *p*_adj_ <0.01 and |logFC| > 0.5 as screening criteria.

**TABLE 1:** The basic information of subjects in the study.

Characteristics	No. of patients (%)		LTA expression (no. of patients)	
			Low (*N* = 260)	High (*N* = 261)
Age (years)	≤65	280 (53.7)	137 (48.9)	143 (51.1)
	>65	241 (46.3)	123 (51.0)	118 (49.0)
Stage	Ⅰ	324 (62.2)	146 (45.1)	178 (54.9)
	Ⅱ	50 (9.6)	28 (56.0)	22 (44.0)
	Ⅲ	120 (23.0)	70 (58.3)	50 (41.7)
	Ⅳ	27 (5.2)	16 (59.3)	11 (40.7)
Grade	G1	96 (18.4)	44 (45.8)	52 (54.2)
	G2	115 (22.1)	62 (53.9)	53 (46.1)
	G3	301 (57.8)	148 (49.2)	153 (50.8)
	High grade	9 (1.7)	6 (66.7)	3 (33.3)
Histological type	Endometrioid	388 (74.5)	187 (71.9)	201 (38.6)
	Serous	111 (21.3)	60 (11.5)	51 (9.8)
	Mixed	22 (4.2)	11 (2.1)	11 (2.1)
Diabetes	No	296 (56.8)	137 (46.3)	159 (53.7)
	Yes	105 (20.2)	56 (53.3)	49 (46.7)
	Unknown	120 (23.0)	67 (55.8)	53 (44.2)
Microsatellite status	MSS	304 (58.3)	173 (56.9)	131 (43.1)
	MSI-L	43 (8.3)	14 (32.6)	29 (67.4)
	MSI-H	159 (30.5)	66 (41.5)	93 (58.5)
	Unknown	15 (2.9)	7 (46.7)	8 (53.5)
Recurrence or metastasis	No	387 (74.3)	182 (47.0)	205 (53.0)
	Yes	77 (14.8)	43 (55.8)	34 (44.2)
	Unknown	55 (10.6)	33 (60.0)	22 (40.0)
Radiation	No	277 (53.2)	145 (52.3)	132 (47.7)
	Yes	220 (42.2)	100 (45.5)	120 (54.5)
	Unknown	24 (4.6)	15 (62.5)	9 (37.5)
Immune cluster	Immune-Low	213 (40.9)	173 (81.2)	40 (18.8)
	Immune-Medium	204 (39.2)	82 (40.2)	122 (59.8)
	Immune-High	104 (20.0)	5 (4.8)	99 (95.2)
Survival status	Alive	436 (83.7)	205 (47.0)	231 (53.0)
	Dead	85 (16.3)	55 (64.7)	30 (35.3)
Survival time		1,134.4 ± 896.5	1,110.4 ± 869.8	1,158.8 ± 921.6

### Cell Culture

The RL95-2 cells (human UCEC cell) were obtained from the Central Laboratory of Central South University Xiangya and cultured with RPMI 1640 (HyClone, Thermo Scientific, Logan, UT) containing 10% fetal bovine serum (FBS) (Gibco, United States), 100 U/ml penicillin, and 100 μg/ml streptomycin (Gibco, United States) in a 37°C cell culture incubator providing 5% CO_2_.

### ssGSEA Estimation and Tumor Microenvironment

The single-sample gene set enrichment analysis (ssGSEA, http://software.broadinstitute.org/gsea/msigdb/index.jsp) is one of the popular and novel enrichment algorithms in recent years, which was extensively utilized to assess patient immune microenvironment in medical studies (Liu et al., 2021a; Liu et al., 2021b; Liu et al., 2021c). In this study, ssGSEA algorithm was used to thoroughly explore the enrichment level of 23 TILs in each sample with UCEC, and the “hclust” package was utilized to perform hierarchical clustering based on them (Barbie et al., 2009; Langfelder and Horvath, 2012). Then, the ESTIMATE method was applied to calculate the abundance of tumor purity and immune and stromal components, and the difference of their levels among the three clusters was examined by Wilcoxon signed-rank test (Yoshihara et al., 2013).

### Identification of Differentially Immune-Related Expressed Genes and Functional Enrichment Analysis

The “Limma” package was used to identify the shared DEIGs associated with the immune microenvironment and tumor mutation burden. These DEIGs were identified by Venn analysis, and their annotations and functional enrichment analysis on Gene Ontology (GO) and Kyoto Encyclopedia of Genes and Genomes (KEGG) were performed by “clusterProfiler” and “enrichplot” packages, respectively. A *p*-value < 0.05 was considered as a threshold.

### Exploration of Signatures Associated With Prognosis

Univariate and multivariate Cox proportional hazards regression analyses were carried out to identify those DEIGs associated with the prognosis of UCEC patients. Those signatures with a significant difference (*p* < 0.05) were recognized as an independent index for prognosis.

### Analysis of Tumor-Infiltrating Immune Cells and Immune Checkpoint Inhibitors

To explore the relationship of this signature and tumor-infiltrating immune cells (TICs), the ssGSEA and CIBERSORT (http://cibersort.stanford.edu/) were utilized to calculate the abundance of TICs of individuals. TIMER (https://cistrome.shinyapps.io/timer/) and TISIDB (http://cis.hku.hk/TISIDB/) verified the correlation between LTA and TILs, immunomodulators (including immunoinhibitory, immunostimulatory, and MHC molecules), chemokines, etc. Their correlation and differentiation were analyzed by Spearman analysis and Wilcoxon signed-rank test, respectively.

TISIDB and TIMER are both integrated repository portals for tumor-immune system interactions; they use different algorithms to evaluate the correlation between the expression of genes and the level of immune cell infiltrations, and then assess their tumor immune status and clinical characteristics. In our present study, we confirmed LTA expression and its relationship with overall survival, and explored the correlation between LTA and immune cell infiltrations by the TIMER and TISIDB.

### LTA Overexpression and Cell Proliferation

LTA lentivirus was constructed in Shenggong Biological Engineering (Shanghai) Co., Ltd, whose overexpression efficiency was detected by immunoblotting (Affnity, DF6453). CCK8 assay (GK10001, GLPBIO) was applied to detect the effect of LTA on cell proliferation. A total of 2,000 cells per well were seeded into 96-well plates, which contained 100 F06Dl of culture medium in each well. Ten microliters of CCK8 reagent was added into each well at the indicated time. The plates were shocked for 20 s and incubated at 37°C for 2 h. Lastly, we measured the OD value of each hole at 450 nm.

### Statistical Analysis

All statistical analyses were proceeded by R software with version number v3.5.2 (https://www.r-project.org/), and the packages involved in this process were “hclust”, “Limma”, “ggplot2”, “survminer”, “clusterProfiler”, and “enrichplot”, among others. A *p*-value <0.05 was considered statistically significant.

## Result

### Identification of Three Immune Subtypes in Uterine Corpus Endometrial Carcinoma

**FIGURE 1:**
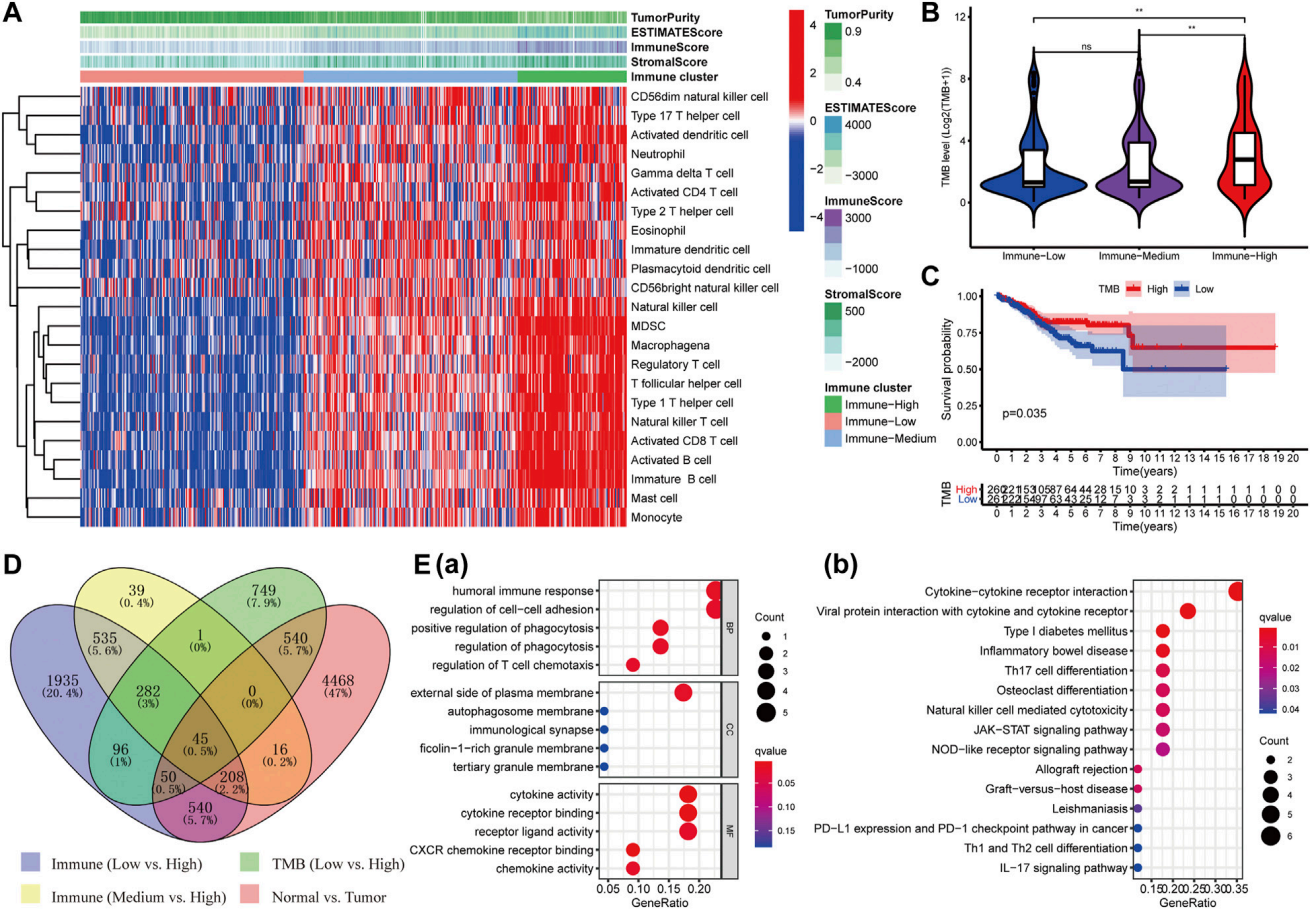
Identification of DEIGs and enrichment analysis. **(A)** Three clusters with distinct immune components (Immune High, Immune Medium, and Immune Low) were obtained based on 23 types of immune cells. The difference of TMB level among the three immune clusters **(B)** and its survival curve in UCEC patients **(C)**. **(D)**Forty-five shared DEIGs were identified according to the four comparison groups as shown in the figure. **(E)** GO **(a)** and KEGG **(b)** analysis of those DEIGs.

The enrichment levels of the common 23 subpopulations of TILs were estimated using the ssGSEA method, which are vital ingredients of the tumor tissue concerning innate and adaptive immunity functions or pathways. We hierarchically clustered the whole TCGA-UCEC cohort and then obtained three different clusters: “Immune-High” (*N* = 104, 19.96%), “Immune-Medium” (*N* = 204, 39.16%), and “Immune-Low” (*N* = 213, 40.88%) (Figure 1A). Subsequently, we assessed stromal (StromalScore), immune (ImmuneScore), and estimate (ESTIMATEScore) scores according to the ESTIMATE algorithm to explore tumor purity (TumorPurity) and heterogeneity of individuals among three clusters. All three scores showed a gradual increasing relationship with statistical differences from “Immune-Low” to “Immune-High” clusters (Wilcoxon signed-rank test, *p* < 0.001) [Supplementary Figures S2A(a–c)], while the tumor purity derived from them had a noticeably opposite trend (Wilcoxon signed-rank test, *p* < 0.001) [Supplementary Figure S2A(d)]. In addition, we further analyzed the levels of TMB among the three clusters, patients in the “Immune-High” cluster had the highest TMB levels compared to “Immune-Medium” (Wilcoxon signed-rank test, *p* < 0.01) and “Immune-Low” clusters (Wilcoxon signed-rank test, *p* < 0.01) (Figure 1B), and the overall survival times of subjects with high TMB were longer than those with low TMB (*p* = 0.035, Figure 1C). Collectively, these results demonstrated that the distinct immune microenvironments could affect the immune status and prognosis of patients with UCEC.

### Extraction of Consensus Differentially Expressed Genes and Enrichment Analysis

Then, we performed differential expression analysis in four comparison groups as shown in Figure 1D and took their intersection. A total of 45 shared targets were identified with the “Limma” package using *p*-value < 0.01 and |logFC| > 0.5 as screening criteria. The top 15 enriched GO terms prominently displayed in the bubble chart were closely related to immunological and tumor-related processes, such as humoral immune response, positive regulation of phagocytosis, and regulation of cell–cell adhesion in biological processes (BP); cytokine activity, CXCR chemokine receptor binding, and cytokine receptor binding in molecular function (MF); and external side of plasma membrane in cellular component (CC) (Figure 1E(a)). The first 15 KEGG pathways mainly involved cytokine–cytokine receptor interaction, viral protein interaction with cytokine and cytokine receptor, Type I diabetes mellitus, and other immune-related pathways (Figure 1E(b)). A total of two genes (LTA and AC004585.1) associated with the prognosis of UCEC were identified using univariate Cox regression analysis (*p* < 0.05, Supplementary Table S1). LTA was ultimately identified as an independent index for prognosis in the multivariate Cox regression analysis [HR = 0.42 (95% CI: 0.22–0.80), *p* = 0.008]. Thus, LTA could be used as a developed biomarker to assess the immune status and prognosis of UCEC patients.

### Exploration of the Relationship Between LTA and Clinicopathological Parameters of UCEC Patients

**FIGURE 2:**
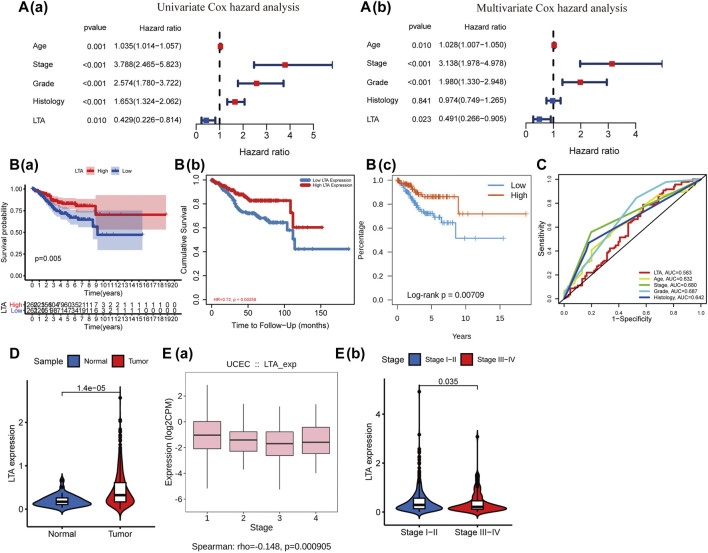
Relationship of LTA and the common clinical features. **(A)** Cox regression analysis of LTA and the common clinical features, and the hazard ratio of LTA was 0.491 with 95% CI (0.266–0.805) (*p* = 0.023) in multivariate Cox hazard analysis. **(B)** Survival curve of LTA, **(a)** Kaplan–Meier survival method, **(b)** TIMER, and **(c)** TISIDB. **(C)** ROC analysis of LTA and the common clinical features. **(D)** The difference of LTA expression in tumor and normal tissues. **(E)** The correlation between LTA expression and tumor stage **(a)** and the distribution of LTA expression in earlier stages (stages I–II) and advanced stages (stages III–IV) **(b)**.

To explore its clinical application and usability, we performed Cox regression analysis on common clinical characteristics, including age, stage, grade, and histological type. The results showed that age (*p* = 0.010), stage (*p* < 0.001), grade (*p* < 0.001), and LTA (*p* = 0.023) could be independent indices for predicting the patients’ overall survival (Figure 2A). The Kaplan–Meier analysis suggested that patients with high LTA expression had better survival prognosis (*p* = 0.005, Figure 2B(a)), which was then verified in TIMER (*p* = 0.00258, Figure 2B(b)) and TISIDB (*p* = 0.00709, Figure 2B(c)). The ROC curve analysis represented a moderate prognostic value for LTA in evaluating the 5-year overall survival of patients with UCEC (AUC = 0.583, Figure 2C). A nomogram was then constructed with LTA and those common clinical traits, and the result indicated that LTA could be a factor affecting patients’ overall survival, while the tumor stage was the biggest factor (*p* < 0.001, Supplementary Figure S2B). Similar to the previous analysis, TIMER indicated that LTA was highly expressed in UCEC compared to normal endometrial tissue (Figure 2D, Supplementary Figure S2C). The finding that TISIDB showed LTA expression was negatively correlated with tumor stages (Spearman, *p* < 0.001, Figure 2E(a)) was consistent with the result that its expression was significantly higher in patients with early stage than those with advanced stage (log-rank test, *p* = 0.035, Figure 2E(b)). These above findings all suggested that LTA could be used as an easily assayed and idiocratic biomarker of patients with UCEC.

### Effects of LTA on the Tumor Microenvironment and Immune Infiltration

**FIGURE 3:**
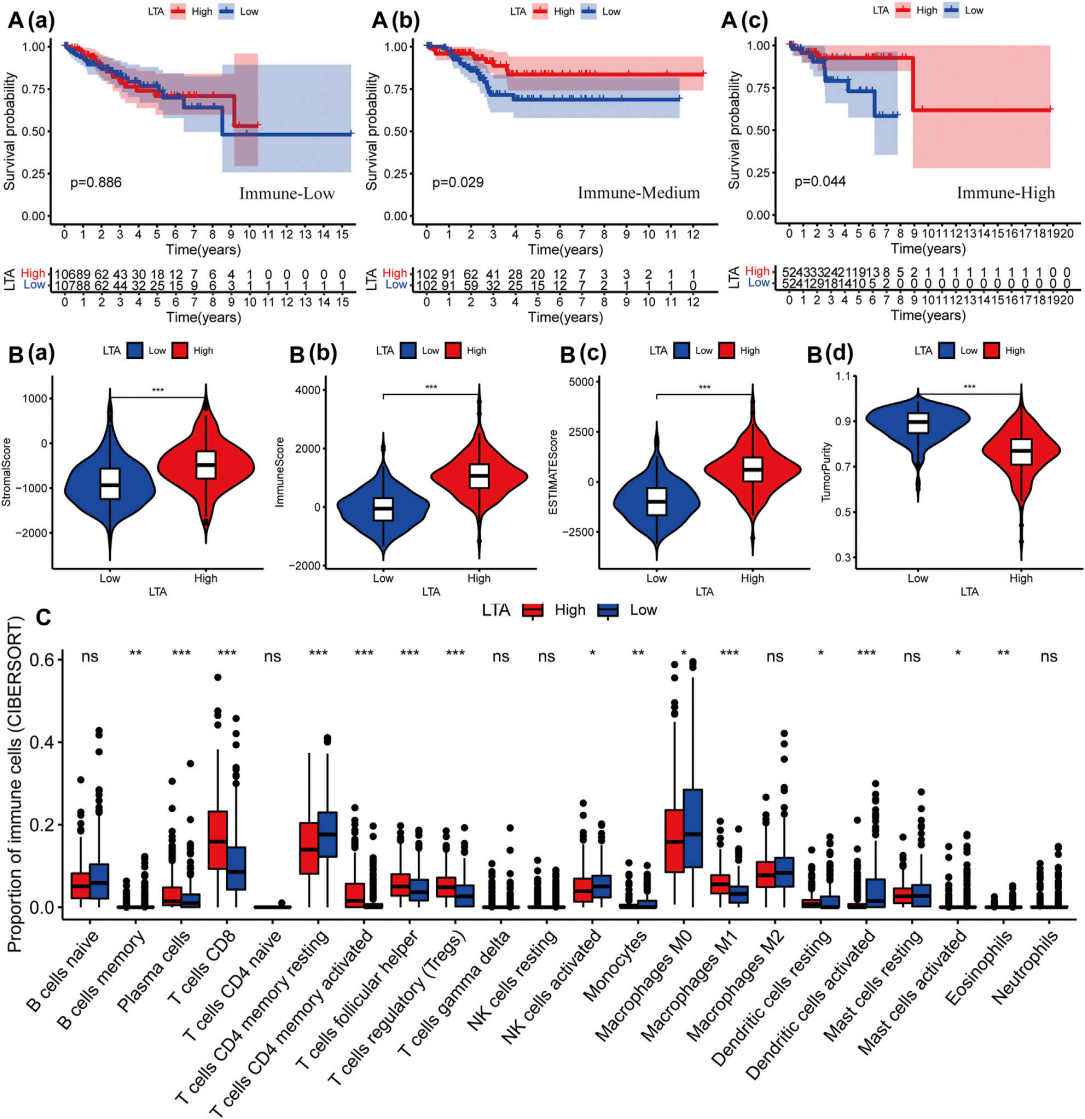
Effect of LTA on immune characteristics. **(A)** The effect of LTA on the survival outcome of patients in three immunotherapy cohorts, “immune-Low” **(a)**, “immune-Medium” **(b)**, and “immune-High” **(c)**. The difference of TME **(B)** and TILs **(C)** between high-LTA and low-LTA expression groups. The *p*-values were labeled by asterisks (ns, no significance, *p < 0.05, **p < 0.01, ***p < 0.001).

Subsequently, we explored the impact of LTA on the survival outcomes in three immunotherapy cohorts; patients with high LTA expression had better prognoses in “Immune-Medium” (*p* = 0.029) and “Immune-High” (*p* = 0.044) cohorts, while there was no statistical difference in the “Immune-Low” (*p* = 0.886) cohort (Figure 3A). Additionally, several recent studies have suggested that the abundance of TICs within the TME could predict phases of tumor inflammation and prognosis and could be related to the expression of immune checkpoint (Pfannstiel et al., 2019). Thus, we analyzed the impact of LTA on the immune microenvironment, which is a vital factor in the occurrence and development of tumors. We stratified the cohort into high-LTA (*N* = 261) and low-LTA (*N* = 260) groups using the median score as a cutoff, the StromalScore, ImmuneScore, and ESTIMATEScore were all increased with statistically significant differences in the high-LTA group, while the TumorPurity was contrary to their trend (Wilcoxon signed-rank test, *p* < 0.05, Figure 3B). We evaluated the differences of 23 types of TILs between high- and low-LTA groups in UCEC patients using the ssGSEA algorithm and Wilcoxon rank-sum test. As expected, all TICs showed higher expression in high-LTA groups (Supplementary Figure S3A), and likewise, TISIDB also indicated that LTA was positively correlated with most tumor-infiltrating immune cells (TIICs) in UCEC (Supplementary Figure S3B). Then, we used the CIBERSORT method to explore the differences of 22 newly defined TICs, 15 types of TICs had significant statistical differences, such as CD8 T cells, memory T cells, and M1 macrophages (Figure 3C). M1 macrophages secrete pro-inflammatory cytokines and chemokines, and present antigens participating in the positive immune response, implying that LTA could affect the human immune microenvironment by regulating the activation state of macrophages.

### Evaluation of the Effect of LTA on Immunological Status in UCEC Using the TIMER

**FIGURE 4:**
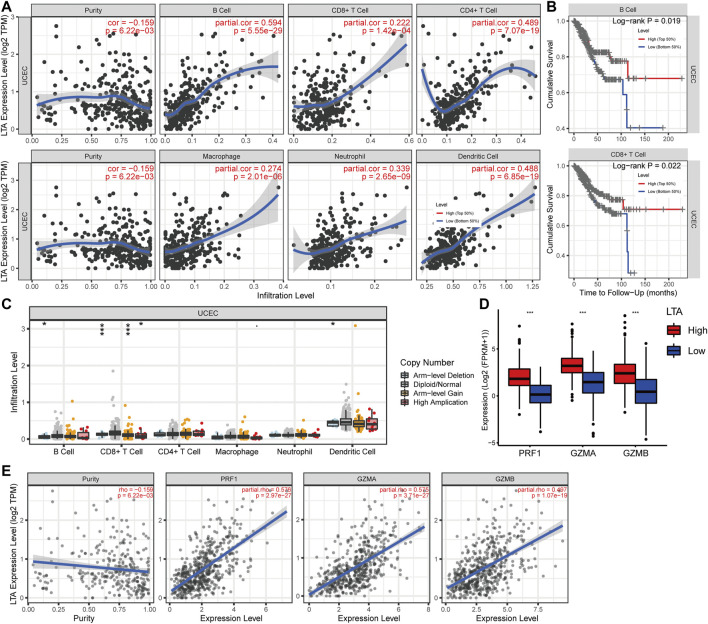
Analysis of the correlation of LTA expression with TICs in UCEC using TIMER. **(A)** Six types of them were positively correlated with the expression of LTA, including B cell, CD8^+^ T cell, CD4^+^ T cell, macrophage, neutrophil, and dendritic cell. **(B)** B cell and CD8^+^ T cell were significantly associated with prognosis of UCEC patients. **(C)** The impact of LTA on patients’ somatic copy number (SCNA) alteration. **(D,E)** The relationship between LTA and the newly immune infiltration biomarkers (PRF1, GZMA and GZMB). The *p*-values were labeled by asterisks (ns, no significance, *p < 0.05, **p < 0.01, ***p < 0.001).

Subsequently, we explored the correlations between LTA and the immune cells in TIMER, its expression was positively correlated with six types of immune cells after adjustment by tumor purity in the TIMER database, namely, B cell, CD8^+^ T cell, CD4^+^ T cell, macrophage, neutrophil, and dendritic cell (Figure 4A). Among them, B cell and CD8^+^ T cell were bound up with the overall survival of patients with UCEC (Figure 4B and Supplementary Figure S4). The copy number of LTA had a significant difference in CD8+ T-cell distributions with different somatic copy number alteration (sCNA) states (Figure 4C). To reflect more fully the relationship between LTA and immune infiltration, we analyzed the correlations between LTA and multiple immune markers characterized by immune cells, 40 out of 45 markers were significantly correlated with LTA expression after adjusting for tumor purity, indicating the magnitude of LTA in tumor immunity regulation (Table 2). Also, we explored the expression level of perforin 1 (PRF1), granzyme A (GZMA), and granzyme B (GZMB), which belonged to the cytolytic molecules in T cells and represents immune infiltration and immune cytolytic activity (Arias et al., 2017). They all showed higher expression in the high-LTA group and were positively correlated with LTA expression (Figures 4D,E).

**TABLE 2:** Correlation analysis between LTA and immune cell gene markers in TIMER.

Description	Gene markers	None		Tumor purity	
		Cor	*p*	partial.cor	partial.*p*
B cell	CD19	0.473	***	0.472	***
	MS4A1	0.620	***	0.633	***
	CD79A	0.615	***	0.582	***
CD8^+^ T cell	CD8A	0.655	***	0.597	***
	CD8B	0.396	***	0.323	***
	IL2RA	0.623	***	0.550	***
Tfh	CXCR3	0.745	***	0.728	***
	CXCR5	0.638	***	0.654	***
	ICOS	0.723	***	0.705	***
Th1	IL12RB1	0.697	***	0.665	***
	CCR1	0.441	***	0.453	***
	CCR5	0.705	***	0.679	***
Th2	CCR4	0.522	***	0.481	***
	CCR8	0.442	***	0.421	***
	HAVCR1	0.185	***	0.175	*
Th17	IL21R	0.706	***	0.662	***
	IL23R	0.182	***	0.203	**
	CCR6	0.423	***	0.414	***
Treg	FOXP3	0.655	***	0.644	***
	NT5E	0.069	0.109	−0.005	0.932
	IL7R	0.404	***	0.363	***
T cell exhaustion	PDCD1	0.600	***	0.555	***
	CTLA4	0.647	***	0.579	***
	LAG3	0.605	***	0.575	***
M1 Macrophage	NOS2	0.115	*	0.068	0.249
	IRF5	0.230	***	0.271	***
	PTGS2	−0.144	**	−0.159	*
M2 Macrophage	CD163	0.395	***	0.343	***
	MRC1	0.399	***	0.403	***
	CD209	0.401	***	0.405	***
TAM	CCL2	0.384	***	0.345	***
	CD86	0.573	***	0.587	***
	CD68	0.501	***	0.472	***
Monocyte	CD14	0.450	***	0.386	***
	CD33	0.527	***	0.459	***
	ITGAX	0.575	***	0.586	***
Natural killer cell	B3GAT1	0.071	0.096	0.031	0.597
	KIR3DL1	0.378	***	0.387	***
	CD7	0.653	***	0.649	***
Neutrophil	FCGR3A	0.431	***	0.402	***
	CD55	−0.104	#	−0.082	0.160
	ITGAM	0.505	***	0.481	***
Dendritic cell	CD1C	0.393	***	0.346	***
	THBD	0.130	*	0.055	0.347
	NRP1	0.169	***	0.122	#

Annotation: #*p* < 0.05; *p < 0.01; **p < 0.001; ***p < 0.0001.

### Analysis of the Relationship Between LTA and Tumor Mutation Burden

**FIGURE 5:**
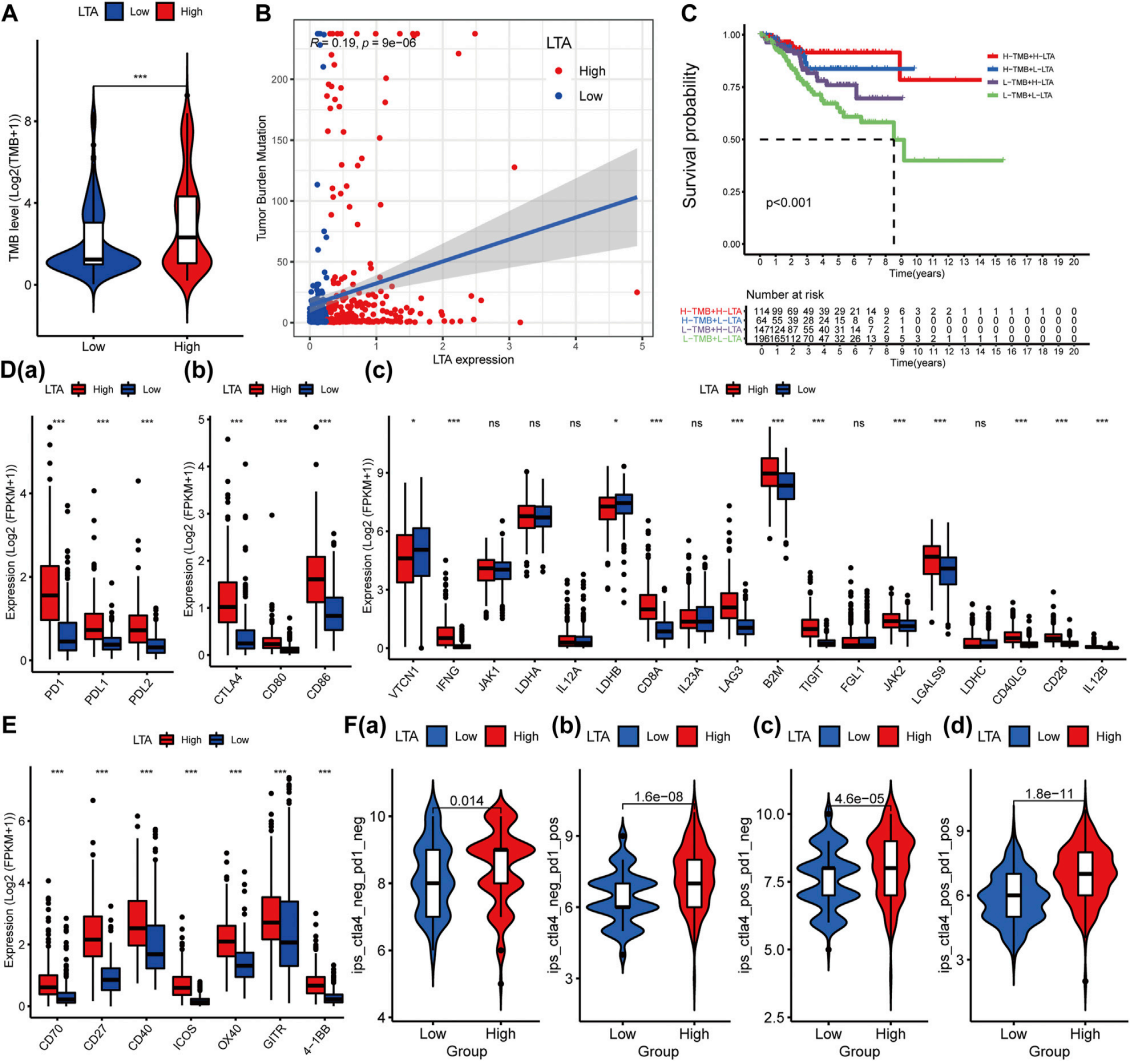
Influence of LTA on TMB and immunoregulatory targets’ expression in clinical trials for patients with UCEC. The difference of TMB in high- and low-LTA groups **(A)**, their correlation **(B)**, and their impact on the overall survival of the patient **(C)**. Effect of LTA on immunoregulatory targets’ expression such as PD1 **(D(a))**, CTLA4 **(D(b))**, and others **(D(c))**, and agonists of T-cell activation **(E)**. **(F)** Immunotherapy score of low- and high-LTA groups based on the TCIA database. The *p*-values were labeled by asterisks (ns, no significance, **p < 0.01, ***p < 0.001).

Many explorations have shown that high TMB could stimulate the patient’s anti-tumor immune response by producing many neoantigens, and thus a longer survival period was achieved (Gubin et al., 2015; Sholl et al., 2020). In our study, patients in the high-LTA group were more inclined to higher TMB levels, which had a significant difference (*p* < 0.001, Figure 5A). There was a positive correlation between the levels of TMB and LTA in patients with UCEC (Spearman, *R* = 0.19, *p* = 9E-06, Figure 5B). Noticeably, patients with the combination of low LTA and low TMB had remarkably poor overall survival than other groups (*p* < 0.001, Figure 5C). Moreover, considering the significant correlation between LTA and TMB, we performed Cox analysis and determined common clinical features; the result indicated that TMB [HR = 0.993 (95% CI: 0.983–0.999), *p* = 0.027] could be an independent factor for predicting the prognosis of UCEC patients (Supplementary Figure S5).

### Evaluation of Sensitivity to Immunotherapy

Immune checkpoint testing is a reliable way to evaluate the patient’s response to immunotherapy, which was considered as first-/second-line therapy in various malignancies because of its long-lasting traits and survival benefits (Derks, 2021; Lucibello et al., 2021; Marin-Acevedo et al., 2021). To assess the effect of LTA expression on immunotherapy, we evaluated the difference of the common immune markers between high- and low-LTA groups, including programmed cell death 1 (PDCD1, best known as PD1), programmed cell death-ligand 1 (PDL1/CD274), and cytotoxic T lymphocyte antigen 4 (CTLA4). The result demonstrated that most of them were up-expression in the high-LTA group, meaning patients with high LTA expression were more likely to get a better immunotherapy response (Figures 5D,E). Then, we explored the immunotherapy effect of CD274/PDL1 and PD1 checkpoint inhibitors based on the TCIA database. The results also indicated that patients with high LTA expression had better immunotherapy response when using the single-CD274/PDL1 inhibitor, or the single-PD1 inhibitor, or CD274/PDL1 in combination with PD1 (Figure 5F).

### Evaluation of the Influence of LTA on Immunotherapy in UCEC Using the TISIDB

**FIGURE 6:**
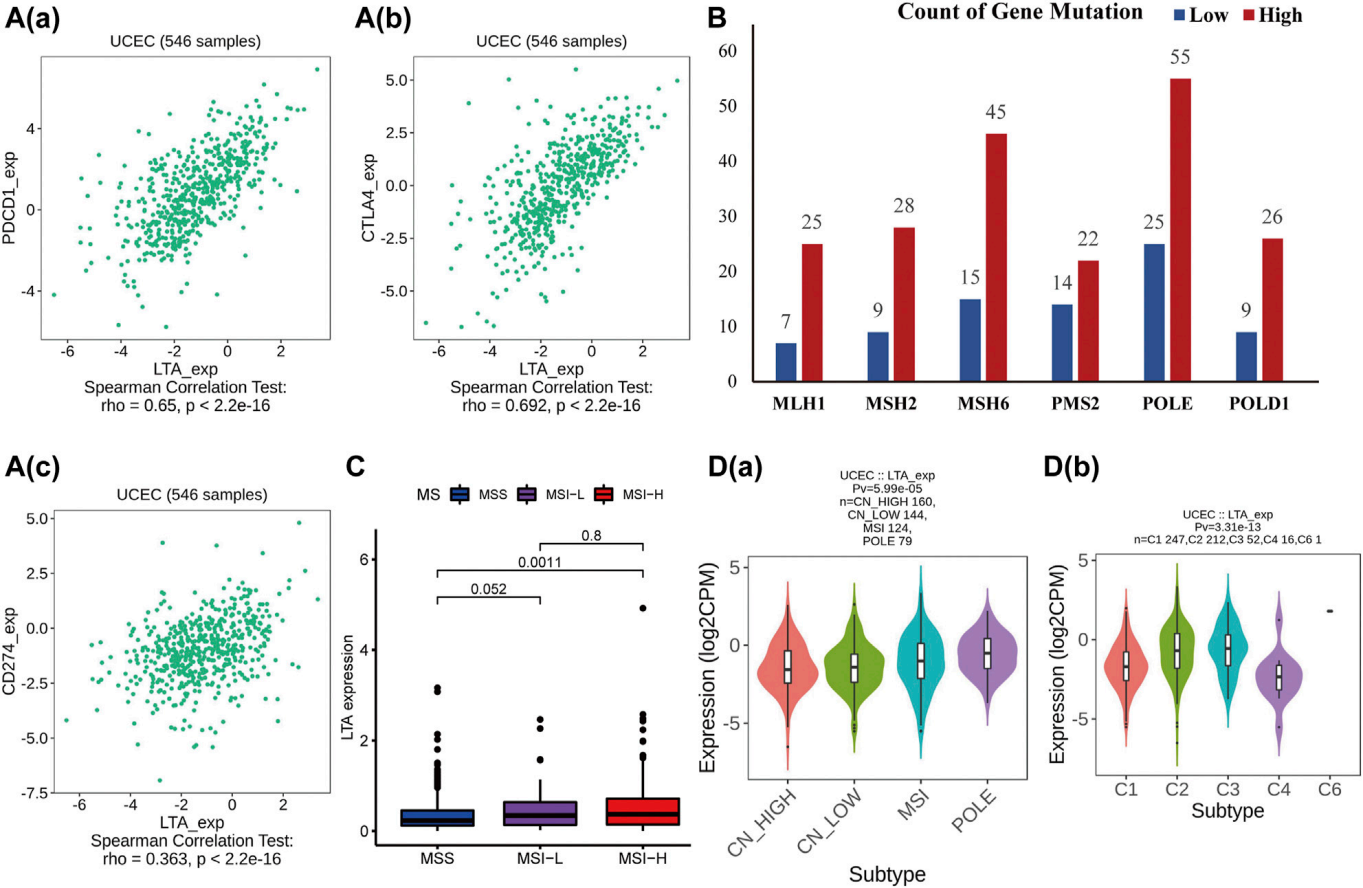
Analysis of the relationships of LTA on immune status using TISIDB. **(A)** The correlation between LTA and PDCD1/PD1 **(a)**, CTLA4 **(b)**, and CD274/ PDL1 **(c)**. **(B)** Gene mutations associated with mismatch repair and POLE proofreading domain between high- and low-LTA groups. **(C)** Differential expression of LTA in three distinct microsatellite states. **(D)** The relationship of LTA on immune **(a)** and molecular **(b)** subtypes, respectively.

The TISIDB was utilized to further investigate the relationship between LTA and common immunomodulators. As shown in Figure 6A and Supplementary Figure S6, LTA was positively correlated with most of them [including several immune checkpoints (PDCD1, CD274, and CTLA4), immune stimulators and receptors, chemokines, and MHC-s] in UCEC. Patients in the high-LTA group had more gene mutations concerning mismatch repair and POLE proofreading domain (such as MLH1, MSH2, MSH6, PMS2, POLE, and POLD1) (Figure 6B). Those were gathered more in the high microsatellite instability (MSI-H) group than in the microsatellite stability (MSS) group (*p* = 0.0011, Figure 6C). We then analyzed LTA expression in different molecular and immune subtypes using TISIDB, which both exhibited significant differences (*p* < 0.05, Figure 6D), which once again demonstrated that patients with high LTA expression had a better response to immunotherapy if they proceeded.

### Verification of the Relationship Between LTA and Immune Status and Exploration of its Effect on the UCEC Cell Proliferation

**FIGURE 7:**
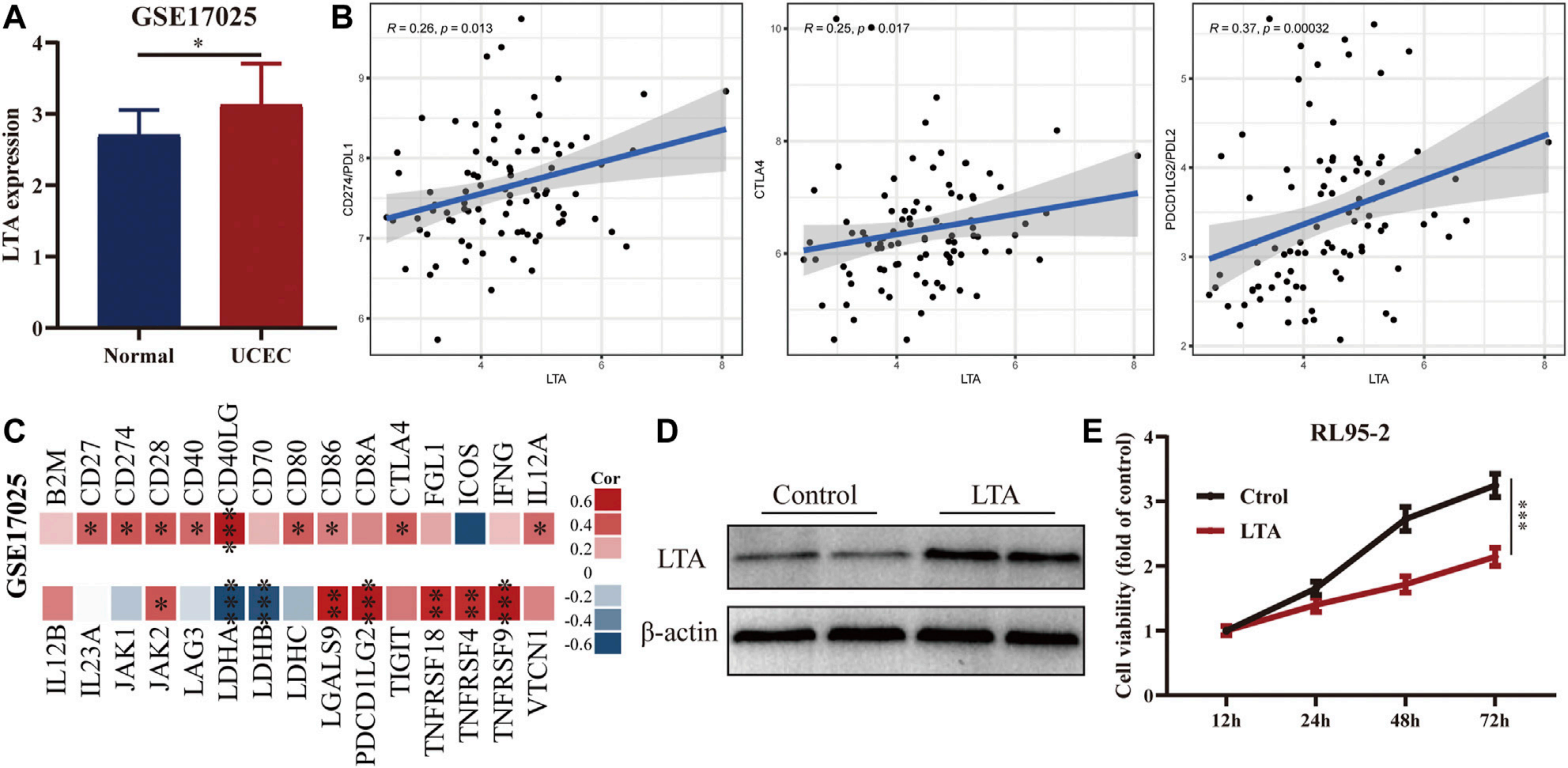
Exploration of the differential expression of LTA in normal and early UCEC patients **(A)** and its correlation with common immune markers **(B,C)** using GSE17025 data. Immunoblotting **(D)** and CCK8 **(E)** analyses of LTA overexpressed in RL95-2 cells (*p < 0.05, **p < 0.01, ***p < 0.001).

To confirm the availability of the appeal study, we explored the differential expression of LTA in normal and early UCEC patients and its correlation with common immune markers using GSE17025 data. LTA was highly expressed in early UCEC samples and has significant correlations with multiple common immune markers (including PDL1 and CTAL4), which are consistent with our previous findings (Figures 7A–C). In addition, we overexpressed LTA in RL95-2 cells (a human endometrial cell) to explore whether it is sufficient to affect cell phenotype. The results indicated that LTA ectopic expression weakened the proliferation ability of RL95-2 cells (Figures 7D,E).

## Discussion

A growing amount of research confirms that those patients with higher TMB levels and more immune components in tumor microenvironment were closely related to superior benefits from immunotherapy in various cancers (Rooney et al., 2015; Liu et al., 2020). In this study, we attempted to identify the potential biomarkers that were differentially expressed between normal and tumor tissues and involved in prognosis, immune microenvironment, and TMB of UCEC patients. LTA was finally substantiated to be significantly upregulated in tumor tissues (especially in the early stage) and closely associated with prognosis and immunological status, so it may be an exploitable biomarker for evaluating the status of UCEC patient’s immunity and the benefits from immunotherapy, and predicting the overall survival outcomes in clinical patients.

In this manuscript, the ssGSEA method was applied to calculate the enrichment scores of every TIL in each sample, and patients were then divided into three clusters based on these scores with distinct TMB levels. Samples with high TMB were more gathered in “Immune-high” cluster with significant statistical differences compared with other clusters and had better overall survivals, indicating that TMB had the potential to predict patients’ prognosis, which dovetailed with previous studies. Then, we identified 45 DEIGs that were differentially expressed between normal and tumor tissues and involved in the immune microenvironment and TMB of UCEC patients. GO and KEGG analyses indicated that most of them were involved in immune-related processes, such as cytokine–cytokine receptor interaction, Th17 cell differentiation, natural killer cell-mediated cytotoxicity, and PD-L1 expression and PD-1 checkpoint pathway in cancer. Afterwards, lymphotoxin alpha (LTA) was identified to be significantly associated with prognosis based on Cox regression analyses. In multiple Cox regression analyses, the hazard ratio of LTA expression was 0.42 with 95% CI (0.22–0.80) (*p* = 0.008). Studies show that LTA is a member of the tumor necrosis factor family and a cytokine produced by lymphocytes. It mediates a large variety of inflammatory and immunostimulatory, and is also an important factor for human B-cell activation and T-cell infiltration (Fu et al., 1998; Ngo et al., 1999). Werner et al. (2021) demonstrated that the loss of LTA-expressed memory B cells was positively correlated with metastasis of melanoma, indicating that it mediated B-cell activation, which is an invaluable element in tumor progression.

More studies reported that immune microenvironment was a vital factor in tumor progression and metastasis (Koelwyn et al., 2017). CIBERSORT algorithm was used to explore the difference and correlation of LTA on the proportions of immune cells, and the results indicated that 15 of them were determined to have significant statistical differences between high- and low-LTA expression groups, namely, memory B cells, plasma cells, CD8 T cells, CD4 memory resting T cells, CD4 memory activated T cells, follicular helper T cells, regulatory T cells (Tregs), activated NK cells, monocytes, M0 macrophages, M1 macrophages, resting dendritic cells, activated dendritic cells, activated mast cells, and eosinophils. For instance, when LTA expression increased, macrophages were mainly in the M1-type polarization state, thereby achieving human immune activation and participating in the positive immune response; these mean that a close but unclear regulatory relationship has existed between them. Then, combining the results with TIMER database analysis, B cells and CD8^+^ T cells were significantly correlated with the expression of LTA, and were intimately associated with prognosis of UCEC, which was consistent with previous research (Mapara et al., 1994; Kulmburg et al., 1998). Several studies demonstrated that tumors with CD8^+^ T-cell infiltration can benefit from immunotherapy to prolong survival (Chen and Flies, 2013; Chen et al., 2019; Hollern et al., 2019; Li et al., 2021). In our study, LTA was positively correlated with CD8^+^ T-cell infiltration and its markers’ expression after adjusting by tumor purity, such as CD8A, CD8B, and IL2RA. Meanwhile, intensive infiltration of other immune cells, such as CD4 memory activated T cells and M1 macrophages, had also been reported to be associated with a good prognosis. Conversely, a low density of activated NK cells and M0 macrophages indicates a poor clinical outcome among patients with UCEC. Then, the TISIDB was utilized to further investigate the relationship between LTA and common immunomodulators. LTA was positively correlated with most of them (including several immune checkpoints, immune stimulators and receptors, chemokines, and MHC-s) in UCEC. Patients in the high-LTA group had more gene mutations concerning mismatch repair and POLE proofreading domain, and those were more gathered in the high microsatellite instability (MSI-H) group than in the microsatellite stability (MSS) group. These conclusions were consistent with our research, meaning LTA may affect the development of tumors by regulating immune activity and could be an exploitable biomarker to further study.

Studies have reported that high TMB is associated with the emergence of new antigens that trigger anti-tumor immunity and is an effective metric for predicting response to immunotherapy (Hollern et al., 2019; Khasraw et al., 2020; Valero et al., 2021; Vega et al., 2021). Notably, patients with high LTA expression were exhibited to have a better prognosis, this phenomenon may be related to their higher level of TMB and immune checkpoints (including PD1 and PDL1). Subsequently, we performed Cox analysis and determined common clinical features; the result indicated that TMB [HR = 0.993 (95% CI: 0.983–0.999), *p* = 0.027] was an independent factor for predicting the prognosis of UCEC patients. These results suggest that LTA can be a novel and developable marker to evaluate the immune activity and effectively distinguish patients who benefited from immunotherapy. Besides, there are still many deficiencies in this study. For example, it is only a retrospective analysis, and the results of our study should be further confirmed by prospective multicenter studies. Then, the results obtained from public databases had not been confirmed in the clinic. Moreover, LTA ectopic expression weakened the proliferation ability of RL95-2 cells, while the potential function and mechanism of LTA in regulating immune activity and functional phenotype have not been thoroughly explored.

## Conclusion

Taken together, we have identified the substantial role of LTA in regulating the patients’ immune activity and predicting their overall survival by a variety of bioinformatics methods. Patients with high LTA expression had better survival and apparently immune-activated phenotypes, such as more TMB, stronger immune cell infiltrations, higher expression of immunosuppressive points, and higher immunophenoscore, meaning they had an immunotherapeutic advantage over those with low LTA expression. In other words, decreased LTA expression was related to the weakened anti-tumor immunity, which, in turn, leads to poor prognosis of patients. These findings provide new insights in predicting prognosis and evaluating the benefit of immunotherapy for clinical UCEC patients.

## Data Availability

Publicly available datasets were analyzed in this study. These data can be found here: Publicly available datasets TCGA (https://portal.gdc.cancer.gov/), GSE17025 (https://www.ncbi.nlm.nih.gov/geo/query/acc.cgi), TIMER (http://timer.comp-genomics.org/), and TISIDB (http://cis.hku.hk/TISIDB/) were analyzed in this study.
