# Supplementary material for: Positive Correlation Between LTA Expression and Overall Immune Activity Suggests an Increased Probability of Survival in Uterine Corpus Endometrial Carcinoma
Source: Front Cell Dev Biol. 2022 Jan 28;9:793793. doi: 10.3389/fcell.2021.793793 (PMC8832144; doi:10.3389/fcell.2021.793793)
Supplement: Supplementary file 1 [file DataSheet1.docx]

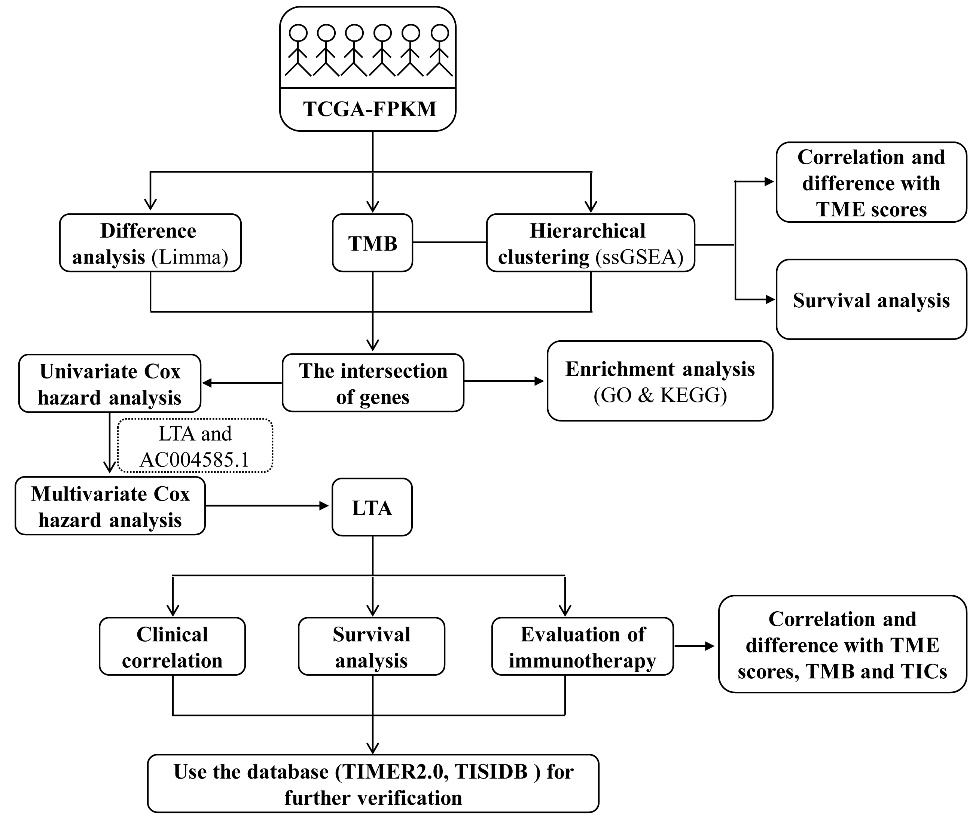


**Figure S1.** A flow diagram demonstrating the study analysis process.

**
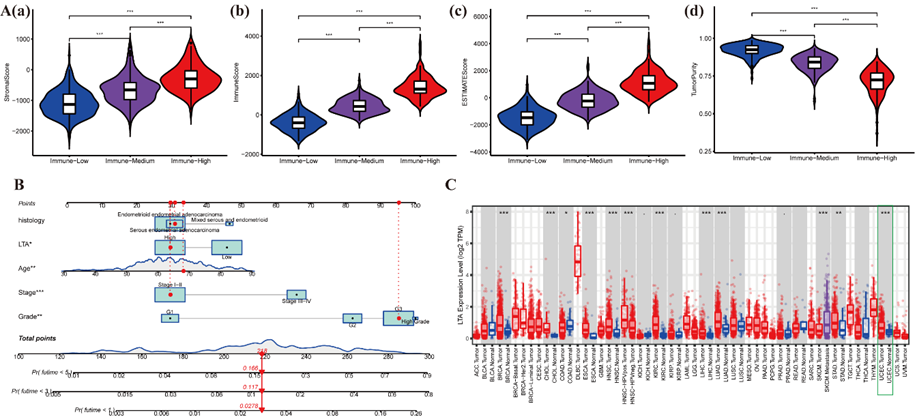
**

**Figure S2.** (A) Exploration of the relationship between the tumor microenvironment and immune clusters. (B) Nomogram predicting the survival probability of 1-, 3-, and 5-year of UCEC patients. (C) Differential expression of LTA in TIMER.


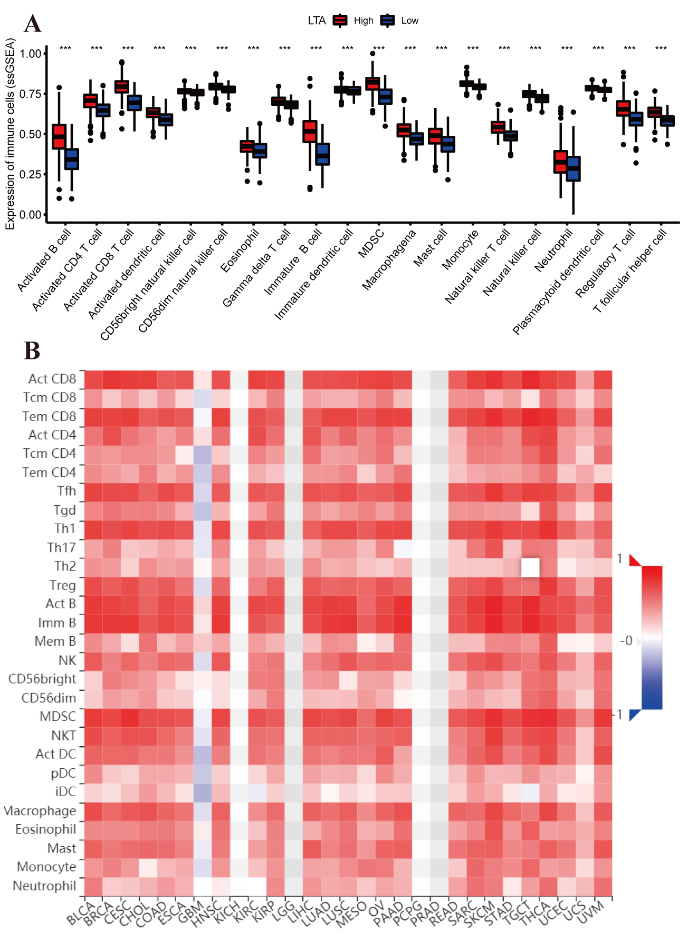


**Figure S3.** Analysis of the relationship of LTA and Immune infiltrating cells by CIBERSORT methods (A) and TISIDB (B).


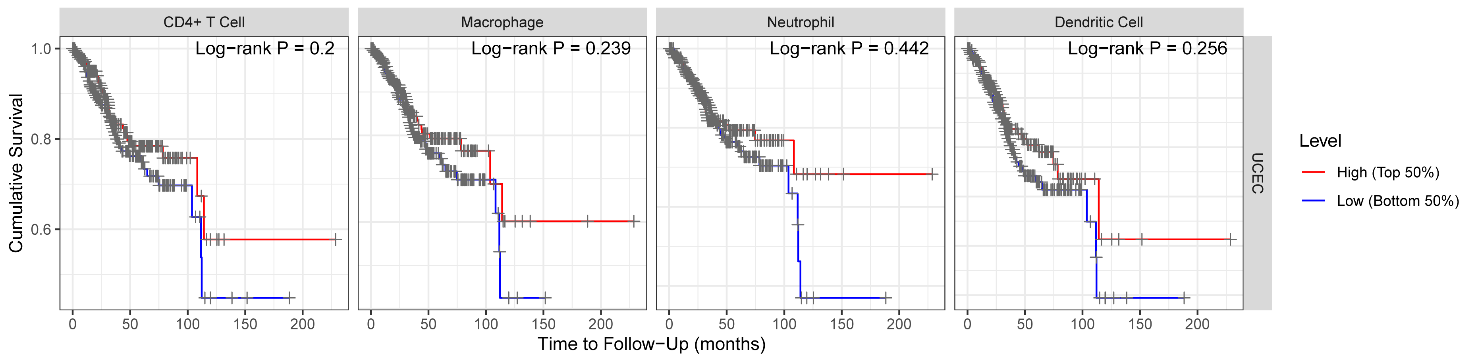


**Figure S4.** Survival analysis of tumor immune infiltrating cells in TIMER exhibiting there were no significant differences (P > 0.05).


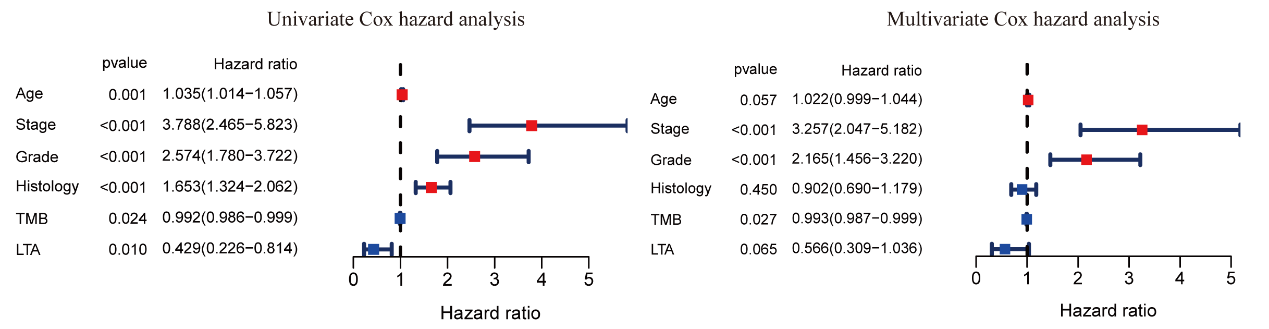


**Figure S5.** Cox regression analysis of LTA, TMB and the common clinical features.


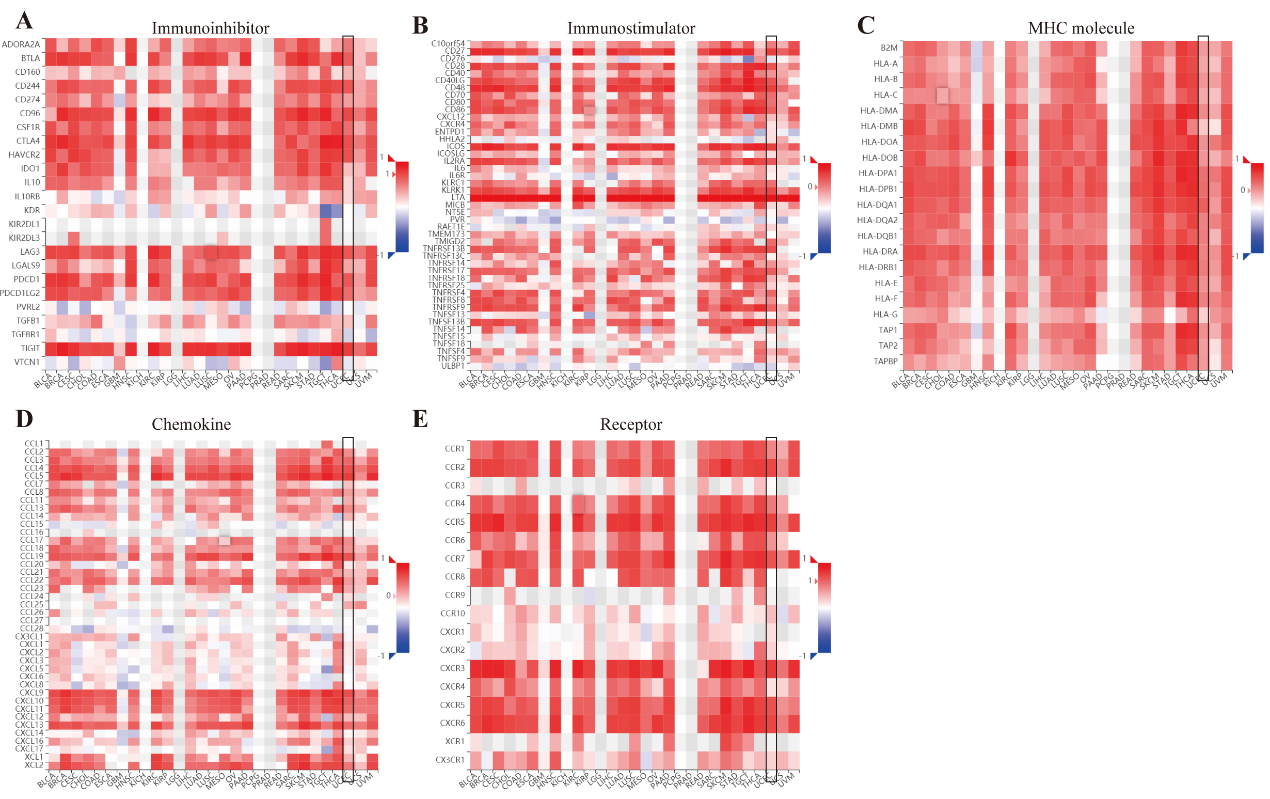


**Figure S6.** Analysis of the relationship of LTA and Immunomodulator using TISIDB database.

**Table S1. Univariate and multivariate Cox regression analysis to identify the prognostic DEIGs.**

| **Method** | **Gene** | **HR** | **95%CI (Low)** | **95%CI (High)** | **p-value** |
| --- | --- | --- | --- | --- | --- |
| **Univariate Cox analysis** | LTA | 0.4226 | 0.2230 | 0.8007 | 8.25E-03 |
|  | AC004585.1 | 0.5730 | 0.3424 | 0.9588 | 3.40E-02 |
| **multiple Cox analysis** | LTA | 0.4226 | 0.2230 | 0.8007 | 8.25E-03 |

Abbreviations: HR, hazard ratio; CI, confidence interval.
